# Crack Cocaine-Induced Cardiac Conduction Abnormalities Are Reversed by Sodium Bicarbonate Infusion

**DOI:** 10.1155/2013/396401

**Published:** 2013-05-23

**Authors:** Carlos Henrique Miranda, Antônio Pazin-Filho

**Affiliations:** Department of Internal Medicine, Division of Emergency Medicine, São Paulo University Medical School at Ribeirão Preto, Rua Bernardino de Campos 1000, 14020-670 Ribeirão Preto, SP, Brazil

## Abstract

We report a dramatic case of a 19-year-old man with crack cocaine overdose with important clinical complications as cardiac arrest due to ventricular fibrillation and *epileptics status*. During this intoxication, electrocardiographic abnormalities similar to those found in tricyclic antidepressant poisoning were observed, and they were reversed by intravenous sodium bicarbonate infusion.

## 1. Introduction

The utilization of crack cocaine is increasing around the world because this drug is inexpensive and possible to smoke. The crack cocaine can cause serious clinical complications during intoxication. There are some descriptions of cardiac arrhythmias, myocardial ischemia and infarction, myocarditis, hypertension, sudden death, seizure, hyperthermia, psychomotor agitation, and so forth [1].

The electrocardiogram helps identify patients at high risk of cardiac and neurologic complications in some types of intoxication as tricyclic antidepressant poisoning, in which a specific electrocardiogram pattern is recognized [2].

We describe a case of crack cocaine overdose with clinical presentation of cardiac arrest due to ventricular fibrillation and *epileptics status* in which we observed an electrocardiographic pattern similar to that found in patients with tricyclic antidepressant poisoning and high risk of complications and these alterations were reversed after sodium bicarbonate infusion.

## 2. Case Presentation

A 19-year-old man ingested large amount of crack cocaine in a suicide attempt. He arrived in the emergency department unconscious and was intubated. After this, he had a cardiac arrest due to ventricular fibrillation reversed with defibrillation. Repeated seizures were observed, and benzodiazepines were administered. 

**Figure 1:**
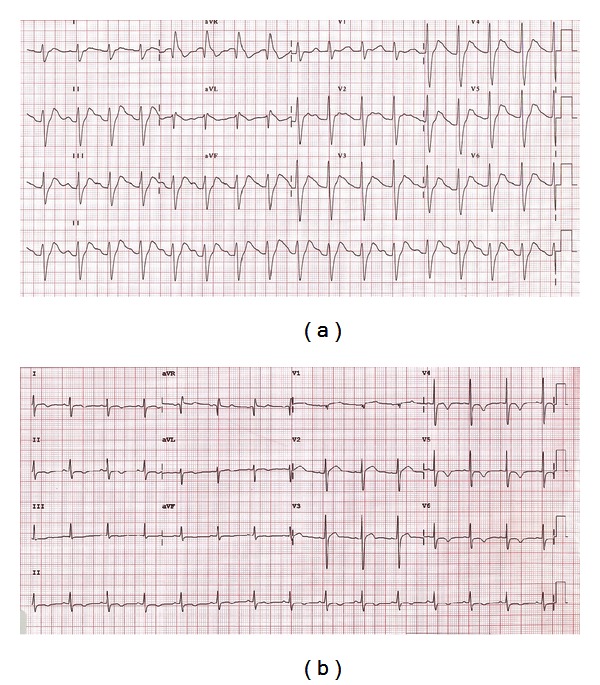
The electrocardiogram (ECG) observed in a 19-year-old man that ingested large amount of crack cocaine (±40 grams) in a suicide attempt. The ECG showed sinus tachycardia with significant widening of QRS (200 ms), significant increase in R wave amplitude in aVR (8.0 mV), QTc interval prolongation (510 ms), and right-axis deviation of the terminal 40 ms vector of the QRS complex in the frontal plane (210°) during the initial evaluation (a), and gradual decrease in QRS duration (100 ms), in R wave amplitude in aVR (4.0 mV), in the QTc interval (428 ms), and in right-axis deviation of the terminal 40 ms vector of the QRS complex in the frontal plane (150°) after intravenous sodium bicarbonate infusion (b).

Electrocardiogram showed sinus tachycardia, prolonged PR and QTc interval (510 ms), widening of the QRS complex (200 ms), and increase in R wave amplitude in aVR (8.0 mV) and right ward of the terminal 40 ms (T40 ms) axis deviation in the frontal plane (210°), Figure 1(a). Blood tests showed combined metabolic and respiratory acidosis (blood pH: 7.10) without other electrolytes disturbances. 

Gradual decreases in QRS duration (100 ms), in QTc interval (428 ms), in R wave amplitude in aVR (4.0 mV), and in the T40 ms axis deviation (150°) were observed after intravenous sodium bicarbonate infusion in a dose of 250 mL × 8.4% and improved ventilation (blood pH: 7.40), Figure 1(b). 

Toxicology screen was positive only for cocaine. Myocardial infarction and any structural cardiac involvement were ruled out because there was no electrocardiographic evidence of myocardial ischemic injury, the dosage of troponin I was only slightly elevated, and the echocardiography was normal. He was discharged without neurological disabilities, and the electrocardiogram was normal.

## 3. Discussion

We described a case of severe crack cocaine overdose with life-threatening ventricular arrhythmias and repeated seizures in which an interesting electrocardiographic pattern similar to that found in tricyclic antidepressant poisoning was observed, and these alterations were completely reversed through the acidosis correction.

The classical electrocardiographic abnormalities observed in acute poisoning by tricyclic antidepressant are the widening of QRS; the increase in R wave amplitude in aVR, the QTc interval prolongation, the right-axis deviation of the terminal 40 ms vector of the QRS complex in the frontal plane, and the Brugada-like pattern. Many studies showed that these alterations can help identify patients with high risk of arrhythmia and seizure, mainly the prolongation of the QRS duration >100 ms. This last alteration predicts a higher risk of complications and is used as a formal indication for systemic sodium bicarbonate administration in many medical services [2]. 

There are many descriptions of reversal of these electrocardiographic alterations through intravenous sodium bicarbonate infusion during tricyclic antidepressant intoxication, and it is recognized as a standard treatment in this condition [3]. These electrocardiographic abnormalities are caused mainly by blocking the fast sodium channels; this effect is called “quinidine-like” because it is similar to that caused by the type I antidysrhythmic drugs. 

The crack cocaine form is manufactured by processing the cocaine with ammonia or sodium bicarbonate to remove the hydrochloride, and in this way it is heat stable which allows it to be smoked [1]. In our case, the patient used this drug in an unusual manner, because he ingested orally the crack cocaine in a suicide attempt. 

 Cocaine can block the fast sodium channels and theoretically trigger similar abnormalities [4]. In this case, the electrocardiographic alterations are probably due to potent sodium channel blockage slowing the cardiac conduction. Although cocaine can block other channels, like the potassium channel, this action is secondary during the clinical presentation, and this drug has a biphasic effect on cardiac repolarization causing a prolongation of the repolarization in a low concentration and a shortening of the repolarization in a high concentration [5]. In our case, the patient ingested a large amount of cocaine, and we observed QTc interval prolongation; however, only the QRS prolongation caused by the sodium blockage was responsible for this alteration, since the JT interval was normal (280 ms) and remained unchanged after bicarbonate administration. 

There are many descriptions of cocaine-related sudden death, but the majority of these patients die before arriving at the hospital, and the mechanisms responsible for these deaths are unclear [1]. Ventricular arrhythmias as ventricular tachycardia and ventricular fibrillation induced by the cocaine may be responsible for it; in our case ventricular fibrillation was observed which was promptly reversed by defibrillation. Wang described one case of a 25-year-old man with cardiac arrest resuscitated with epinephrine after the ingestion of crack cocaine who presented electrocardiographic alterations similar to those showed here that improved after sodium bicarbonate infusion; however, in this case arrhythmias and seizure were not described [6]. 

 These life-threatening ventricular arrhythmias can be precipitated due to myocardial infarction or ischemia induced by the cocaine [1]; but in our case, alterations compatible with acute coronary syndrome were not observed, and echocardiography was normal. Therefore, these alterations appear to be a direct local effect of the cocaine on the heart. 

Acidosis can increase the toxic effect of the cocaine on the heart in that it increases the sodium channel blocking degree and decreases the conduction between the gap junctions, and, because of this, the acidosis correction is an important goal in the treatment of these patients [7]. 

 Some reports have described the treatment of cocaine-induced arrhythmias with the administration of sodium bicarbonate [8]. Jonsson et al. described an interesting case of cocaine overdose complicated by *epileptics status*, accelerated idioventricular rhythm associated with metabolic acidosis, and this arrhythmia was abated through the correction of the acidosis with bicarbonate infusion and ventilation [9]. Kerns et al. reported three cases of cocaine overdose with wide complex tachycardia; in one of these cases the rhythm disturbance resolved without direct treatment, but in the other two cases this arrhythmia resolved after intravenous bicarbonate infusion [10].

 In our case, the cocaine-induced electrocardiographic alterations were reversed through the acidosis correction by sodium bicarbonate intravenous infusion and improved ventilation. Tricyclic antidepressant intoxication was actively ruled out in this case through the negative blood dosage.

There are some descriptions of transient Brugada-like pattern after cocaine and tricyclic antidepressant intoxication which is defined by right bundle branch block associated with coved ST-segment elevation in leads V1 and V2. In our case, we observed important alterations in cardiac conduction but not the Brugada-like pattern [11]. 

Postresuscitative effects on the heart might have been responsible for some of these alterations, although this electrocardiographic pattern is not commonly observed in other situations of cardiac arrest, and in this case the patient was promptly resuscitated because the cardiac arrest happened during the initial medical evaluation. 

The prevalence and prognostic of these electrocardiographic changes during cocaine overdose are unknown, but they could help identify patients at high risk of complications during this clinical setting, and the physicians need to suspect crack cocaine overdose in unconscious patients admitted to the hospital with electrocardiogram pattern compatible with sodium channel blocking. 

## 4. Conclusion

The crack cocaine can induce an electrocardiographic pattern similar to that observed in acute poisoning by tricyclic antidepressants which is reversed by sodium bicarbonate infusion. These alterations may help identify patients at high risk of ventricular arrhythmia and seizure during crack cocaine overdose.
